# Smoking and mental illness: results from population surveys in Australia and the United States

**DOI:** 10.1186/1471-2458-9-285

**Published:** 2009-08-07

**Authors:** David Lawrence, Francis Mitrou, Stephen R Zubrick

**Affiliations:** 1 Centre for Developmental Health, Curtin Health Innovation Research Institute, Curtin University of Technology, Perth, Australia; 2 Telethon Institute for Child Health Research, PO Box 855, West Perth, WA 6872, Australia

## Abstract

**Background:**

Smoking has been associated with a range of mental disorders including schizophrenia, anxiety disorders and depression. People with mental illness have high rates of morbidity and mortality from smoking related illnesses such as cardiovascular disease, respiratory diseases and cancer. As many people who meet diagnostic criteria for mental disorders do not seek treatment for these conditions, we sought to investigate the relationship between mental illness and smoking in recent population-wide surveys.

**Methods:**

Survey data from the US National Comorbidity Survey-Replication conducted in 2001–2003, the 2007 Australian Survey of Mental Health and Wellbeing, and the 2007 US National Health Interview Survey were used to investigate the relationship between current smoking, ICD-10 mental disorders and non-specific psychological distress. Population weighted estimates of smoking rates by disorder, and mental disorder rates by smoking status were calculated.

**Results:**

In both the US and Australia, adults who met ICD-10 criteria for mental disorders in the 12 months prior to the survey smoked at almost twice the rate of adults without mental disorders. While approximately 20% of the adult population had 12-month mental disorders, among adult smokers approximately one-third had a 12-month mental disorder – 31.7% in the US (95% CI: 29.5%–33.8%) and 32.4% in Australia (95% CI: 29.5%–35.3%). Female smokers had higher rates of mental disorders than male smokers, and younger smokers had considerably higher rates than older smokers. The majority of mentally ill smokers were not in contact with mental health services, but their rate of smoking was not different from that of mentally ill smokers who had accessed services for their mental health problem. Smokers with high levels of psychological distress smoked a higher average number of cigarettes per day.

**Conclusion:**

Mental illness is associated with both higher rates of smoking and higher levels of smoking among smokers. Further, a significant proportion of smokers have mental illness. Strategies that address smoking in mental illness, and mental illness among smokers would seem to be important directions for tobacco control. As the majority of smokers with mental illness are not in contact with mental health services for their condition, strategies to address mental illness should be included as part of population health-based mental health and tobacco control efforts.

## Background

Smoking remains one of the leading causes of preventable disease and death both in Australia and the United States [1,2]. Efforts to reduce the prevalence of smoking continue to be of public health significance. The association between smoking and mental illness has been known and studied for many years, but the focus of much of the work in this field has been on people with severe mental illness, such as schizophrenia, or people being treated by psychiatric services [3-8]. For instance, a recent meta-analysis by de Leon and Diaz of 42 individual studies reported that people with schizophrenia had odds of smoking 5.3 (95% CI: 4.9–5.7) times higher than the general population [9].

Glassman *et al *reported an association between smoking and major depression from the St Louis Epidemiological Catchment Area Survey [10]. Lasser *et al *reported the first major population study in the US showing the substantial proportion of smokers who met DSM-III-R criteria for mental disorders, many of whom were not in contact with mental health services [11,12]. They reported that people with DSM-III-R mental illnesses in the month prior to the survey had a smoking rate twice as high as people with no mental illness, and consumed an estimated 44% of cigarettes smoked by adults in the United States.

The excess mortality among people with mental illness due to common conditions for which smoking is a known risk factor, such as cardiovascular disease, respiratory disease and cancers, has been extensively described, from both service-based and community-based samples, and there is evidence of a growing disparity in mortality rates between those with and without mental illness [13-17]. For example, in Western Australia, cardiovascular mortality fell significantly in the general population between 1980–1998, but there was no decline among people with mental disorders [18]. One possible explanation for this phenomenon may be that the public health interventions that have helped to reduce cardiovascular mortality in the general community, such as the efforts that have reduced the prevalence of smoking in both Australia and the US, may have been less effective among people who have mental illness.

It has been hypothesised that one reason for the strong association between mental illness and current smoking is that mental illness is a factor in smoking initiation. Depression and anxiety in teenagers have been found to be strong predictors of smoking experimentation and the transition to daily smoking [19-21]. However, smoking has also been associated with the onset of psychiatric symptoms in teenagers [21,22]. This has suggested that smoking and depressive and/or anxiety symptoms may have onset around the same time, possibly associated with common prior causes [23-25]. However, controlling for common causal factors does not completely remove the predictive ability of smoking on mental illness, particularly depression and anxiety and vice versa. This suggests the possibility that separate causal mechanisms may operate in both directions, in addition to the predictive ability of common causal factors [21,26-28].

The large overlap between mental illness and smoking is not entirely surprising considering the known effects of nicotine on the brain. Nicotine is a psychostimulant that effects several neuroregulators that influence behaviour and mood [29,30]. In some circumstances, nicotine can relieve symptoms of both depression and anxiety [31,32]. Nicotine cessation can also precipitate depressive symptoms, particularly in people with a history of major depression [33,34]. However, it has been questioned whether these are independent depressive symptoms or unpleasant withdrawal symptoms [35,36]. The onset of depressive symptoms following smoking cessation has been linked with lower quit rates, and most commonly occurs in people with depression [37-39]. These factors have lead to the self-medication hypothesis – that smokers with mental illness choose to smoke because it is the easiest, most readily accessible way to control symptoms of mental illness, especially for those who are not receiving any prescribed form of treatment for their mental health condition [40].

While the self-medication theory implies there is a therapeutic benefit to smoking which people with depressive or anxiety symptoms find helpful, research suggests that smoking provides temporary relief from immediate symptoms, while overall creating a greater level of anxiety and stress [31,35,41]. Thus the alleviation of stress and anxiety by smoking may be part of the withdrawal feedback mechanism. It has been suggested that the self-medication hypothesis has too often been used as a justification for not acting to curb cigarette smoking in this population despite the fact that nicotine is not regarded as the most appropriate therapy for any mental health problem, and that even if it were, cigarettes would not be an appropriate form of administering it [37].

The study of Lasser *et al *was based on data from the US National Comorbidity Survey which was collected in 1992. Smoking rates have been in decline in the US since that time [42]. More current data are now available with the release of the public use file from the US National Comorbidity Study-Replication which was conducted between 2001 and 2003, and data from the 2007 Australian Survey of Mental Health and Wellbeing. We hypothesised that despite the decline in overall smoking rates in both the US and Australia since 1992, people with mental illness would continue to represent a disproportionally high number of smokers.

We used data from two recent nationally representative surveys that employed the WHO Composite International Diagnostic Interview (CIDI) to estimate prevalence of mental health disorders among smokers–the National Comorbidity Study-Replication in the US and the 2007 Australian Survey of Mental Health and Wellbeing. We also examined data from the annual US National Health Interview Survey, which has used a short measure of psychological distress as a proxy of severe mental illness, to examine whether it would be feasible to monitor the relationship between mental illness and smoking as part of routine surveillance systems.

## Methods

### Data sources

#### Survey of Mental Health and Wellbeing (SMHWB)

The Australian SMHWB was conducted by the Australian Bureau of Statistics (ABS) between August and December 2007 [43]. It comprised a nationally representative sample of 8,841 adults aged 16–85 years living in private dwellings, drawn using a stratified multistage area-based sample design. The survey was conducted by personal interview. The survey measured the prevalence of three major groups of disorders – anxiety disorders, affective disorders and substance use disorders – using Version 3 of the CIDI [44]. Full details of the survey methodology have been published elsewhere [45].

#### National Comorbidity Study Replication (NCS-R)

The US NCS-R was a nationally representative probability sample of 9,282 individuals conducted by means of personal interviews by the Institute for Social Research at the University of Michigan [46]. The survey measured the prevalence of most DSM-IV and ICD-10 mental disorders using Version 3 of the CIDI. The NCS-R employed a stratified multistage area-based sampling design. A long-form/short-form approach was used with all 9,282 respondents completing part one of the questionnaire covering the core CIDI disorders, while a sub-sample completed part two (n = 5,692), which covered risk factors, consequences, service use and disorders of secondary importance or those that were time-consuming to administer. Of the disorders used in this study, all the affective disorders and all of the anxiety disorders except for post-traumatic stress disorder were included in the part one questionnaire, while the substance use disorders and post-traumatic stress disorder have been estimated using the part two sample. Due to a problem with questionnaire sequencing the diagnosis of obsessive-compulsive disorder could not be extracted from the NCS-R data. Full details of the survey methodology and procedures have been published previously [47,48].

#### National Health Interview Survey (NHIS)

The US NHIS is an annual survey conducted by means of personal interview by the National Center for Health Statistics [49]. The NHIS uses a multistage area-based probability sampling design. The survey has three basic components, the family component, the sample child and the sample adult. The family component collects information on household composition, demographic characteristics and basic health indicators. For each selected family, one child and one adult are selected to complete more detailed questionnaires. For this study, data was taken from the 2007 sample adult component which includes measures of smoking status and level of psychological distress. There were 23,393 sample adults in the 2007 NHIS.

As the study consisted of analysis of publicly available confidentialised files, no institutional ethics approval was required.

### Measures

#### Mental illness

Mental disorders were assessed in the NCS-R and SMHWB using Version 3 of the CIDI [44]. The CIDI is a fully structured interview questionnaire which was administered in both surveys by lay interviewers using computer assisted interviewing software. The CIDI is designed to cover the diagnostic criteria for mental disorders in both the International Classification of Diseases, 10th edition (ICD-10), and the Diagnostic and Statistical Manual of Mental Disorders, 4th Edition (DSM-IV) [50,51]. The CIDI includes an initial screener for major symptoms of mental disorders followed by detailed questions on each disorder. The average interview time in the SMHWB was 90 minutes with the majority of the time taken up by the administration of the CIDI. For this paper we have used the ICD-10 diagnoses rather than the DSM-IV diagnoses as results from the Australian SMHWB have only been released on the ICD-10 basis to date.

In the NHIS, specific mental disorders are not assessed. Instead the NHIS employs a short measure of non-specific psychological distress, the Kessler 6 scale [52,53]. This scale was specifically designed to be sensitive to the upper 90th-99th percentile range of population distribution of mental disorders. Within this range it has high discrimination.

#### Tobacco use

In the NCS-R, current smokers were identified from responses to the question "are you a current smoker, ex-smoker, or have you never smoked?" In the SMHWB, respondents were asked "do you currently smoke every day, at least weekly, less than weekly, or not at all?" In the NHIS, the sample adult was asked "do you now smoke cigarettes every day, some days or not at all?" For consistency with the NCS-R, current smokers were taken as those who smoked daily or less than daily.

#### Service use

In the SMHWB, respondents were given the definition "The next few questions are about problems with your mental health. This includes but is not restricted to such things as stress, anxiety, depression or dependence on alcohol or drugs." Respondents were then asked how often they accessed the following health services for problems with their mental health in the 12 months prior to the survey: general practitioner, psychiatrist, psychologist, mental health nurse or other professional providing specialist mental health services, or other health professional including medical specialist, other professional providing general services or complementary and alternative therapist.

### Weighted estimates and standard errors

Survey weights were applied to calculate estimates of totals and proportions. These weights have been calculated to adjust for potential non-response. For the NCS-R, the supplied weights had been normalised to sum to the sample size, whereas the weights supplied with the SMHWB sum to the population size. We re-weighted the NCS-R data by multiplying the normalised weights by a constant factor so that the weights summed to the population size. This adjustment to the weights has no effect on estimates or standard errors for proportions. Standard errors and confidence intervals for the NCS-R and the NHIS were calculated adjusting for the complex nature of the sample design using expansion in Taylor series [54]. Standard errors and confidence intervals for the SMHWB were calculated using the jack-knife method of replicate weighting [54].

### Modelling the relationship between smoking and psychological distress

The NHIS assessed the level of non-specific psychological distress using the K6 scale, which generates a score between 0 and 24. We used logistic regression modelling to assess the relationship between psychological distress score, and probability of being a smoker. As logistic regression models the log of the odds ratio, we did not enter the K6 score as a linear variable in the model as that would imply an exponential relationship between K6 score and probability of smoking. With no theoretical basis to assume a particular shape for the relationship, we used the generalised additive models (GAM) framework to fit a non-parametric spline curve to describe the relationship [55]. Similarly we used GAMs when using regression to model the average number of cigarettes smoked per day by level of psychological distress. All of the statistical analyses were undertaken using SAS software, Version 9.1 [56].

## Results

### Mental illness and smoking in Australian adults

From the 2007 SMHWB, the ABS estimated that 20% of the adult population had a mental illness in the 12 months prior to the survey (95% CI: 18.9%–21.1%). Of those adults with a mental illness, 1 156 600 were current smokers – a smoking prevalence of 36.2% (95% CI: 32.9%–39.6%), almost double the 18.8% smoking prevalence among adults with no mental illness (95% CI: 17.2%–20.4%). In total, 3 566 800 adults were current smokers in 2007, and people with mental illness represented 32.4% (95% CI: 29.5%–35.3%) of current smokers. In contrast, only 15.6% (95% CI: 29.5%–35.3%) of people who have never smoked had a mental illness in the 12 months prior to the survey.

### Mental illness and smoking in non-institutionalised adults in the US

Basing mental illness diagnosis on ICD-10, and using as similar as possible definitions of disorders as was applied in the Australian study, we estimated that 19.7% of the US adult civilian non-institutionalised population had a mental illness in the 12 months prior to the survey, conducted in 2001–2003 (95% CI: 18.9%–20.6%). Among those adults with a mental illness, 40.1% were current smokers (95% CI: 37.6%–42.7%) which was almost double the 21.3% smoking prevalence in adults with no 12-month mental illness (95% CI: 20.1%–22.5%). People with mental illness represented 31.7% of current smokers (95% CI: 29.5%–33.8%), or 16.1 million people out of an estimated total 51.0 million adult smokers in the US. In contrast, only 15.1% of people who had never smoked had a mental illness in the 12 months prior to the survey (95% CI: 13.8%–16.4%).

In both the US and Australia, smoking rates were highest among those with substance use disorders, where around two-thirds of sufferers were current smokers (Tables 1 and 2). In the US, 45.1% of adults with affective disorders smoked (95% CI: 41.1%–49.2%), and 37.6 of adults with anxiety disorders smoked (95% CI: 34.6%–40.7%), compared with 21.3% of adults with no 12-month mental disorder (95% CI: 20.1%–22.5%).

**Table 1:** Australian adults 16–85 years: Prevalence of mental disorders in the 12 months prior to the survey, and smoking rate, by type of disorder

	Proportion with mental disorder (%)	95% CI	Smoking rate (%)	95% CI
Mental disorders				
Anxiety disorders				
Panic disorder	2.6	2.1 – 3.1	39.6	33.4 – 45.8
Agoraphobia	2.8	2.3 – 3.3	37.0	32.7 – 41.4
Social phobia	4.7	4.1 – 5.3	32.9	29.6 – 36.2
Generalised anxiety disorder	2.7	2.1 – 3.3	45.8	39.0 – 52.7
Obsessive-compulsive disorder	1.9	1.5 – 2.3	41.1	33.1 – 49.2
Post-traumatic stress disorder	6.4	5.8 – 7.0	33.7	30.2 – 37.1
Any anxiety disorder *	14.4	13.5 – 15.3	33.4	31.0 – 35.9
				
Affective disorders				
Depressive episode	4.1	3.5 – 4.7	38.4	33.5 – 43.3
Dysthymia	1.3	1.0 – 1.6	38.3	29.4 – 47.2
Bipolar affective disorder	1.8	1.4 – 2.2	58.1	49.1 – 67.1
Any affective disorder *	6.2	5.5 – 6.9	43.4	39.2 – 47.5
				
Substance use disorders				
Alcohol harmful use	2.9	2.4 – 3.4	43.7	37.7 – 49.7
Alcohol dependence	1.4	1.1 – 1.7	61.3	51.3 – 71.2
Drug use disorder	1.4	1.1 – 1.7	72.6	62.6 – 82.5
Any substance use disorder *	5.1	4.5 – 5.7	53.6	48.8 – 58.5
				
Any mental disorder *	20.0	18.9 – 21.1	36.2	32.9 – 39.6
No mental disorder	80.0	78.9 – 81.1	18.8	17.2 – 20.4
Total persons aged 16–85 years	100.0		22.3	20.9 – 23.7

* Note: As a person may have had more than one disorder in the 12 months prior to the survey, the components will not add to the totals shown.

Source: 2007 Australian Survey of Mental Health and Wellbeing [43].

**Table 2:** US adult civilian non-institutionalised population 18 years and over: Prevalence of mental disorders in the 12 months prior to the survey, and smoking rate, by type of disorder

	Proportion with mental disorder (%)	95% CI	Smoking rate (%)	95% CI
Mental disorders				
Anxiety disorders				
Panic disorder	3.7	3.1 – 4.2	45.2	40.6 – 49.7
Agoraphobia	3.6	3.2 – 3.9	42.0	35.9 – 48.1
Social phobia	7.7	7.0 – 8.3	35.0	30.6 – 39.5
Generalised anxiety disorder	2.7	2.3 – 3.1	45.2	37.9 – 52.5
Obsessive-compulsive disorder	n.a.			
Post-traumatic stress disorder	4.4	3.7 – 5.1	40.0	32.8 – 47.3
Any anxiety disorder *	15.3	14.3 – 15.9	37.8	34.5 – 41.0
				
Affective disorders				
Depressive episode	3.4	3.0 – 3.7	41.3	34.3 – 48.3
Dysthymia	2.4	2.1 – 2.8	45.8	38.5 – 53.0
Bipolar affective disorder	2.5	2.2 – 2.9	50.4	42.8 – 58.0
Any affective disorder *	6.9	6.3 – 7.6	45.1	41.1 – 49.2
				
Substance use disorders				
Alcohol harmful use	2.9	2.4 – 3.5	62.3	55.8 – 68.9
Alcohol dependence	1.4	1.0 – 1.8	70.9	59.6 – 82.3
Drug use disorder	1.3	1.0 – 1.6	67.1	54.3 – 80.0
Any substance use disorder *	3.8	3.1 – 4.6	63.6	56.6 – 70.6
				
Any mental disorder *	19.7	18.9 – 20.6	40.1	37.6 – 42.7
No mental disorder	80.3	79.4 – 81.1	21.3	20.1 – 22.5
Total persons aged 18 years and over	100.0		25.0	23.9 – 26.2

### Age, mental illness and smoking

**Figure 1:**
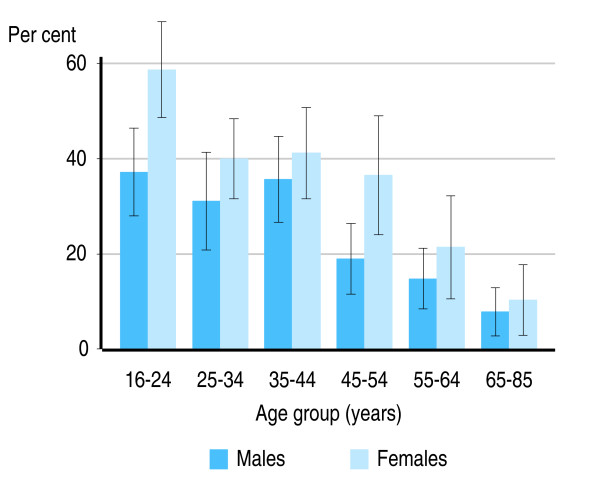
**Proportion of Australian smokers with a mental disorder, by age group and sex**. Source: 2007 Australian Survey of Mental Health and Wellbeing, customised tables [43].

**Figure 2:**
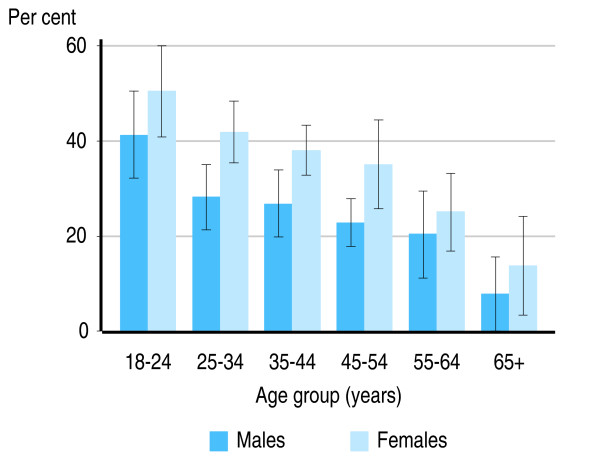
**Proportion of United States smokers with a mental disorder, by age group and sex**. Source: National Comorbidity Survey-replication [46].

In both the US and Australia, smokers aged 16–24 years, who are likely to have started smoking most recently, had the highest rates of mental illness (Figures 1 and 2). In Australia, 37.2% of male smokers aged 16–24 years (95% CI: 28.0%–46.4%) and 58.7% of female smokers in this age group (95% CI: 48.7%–68.5%) had a 12-month mental disorder.

### Age, mental illness, comorbid substance use and smoking

The mental disorders shown in Tables 1, 2 &3 are not mutually exclusive categories. An individual may have more than one disorder. As smoking rates were so high among those with alcohol or drug disorders, we investigated the extent to which comorbid substance use was associated with the rates of smoking for people with anxiety and affective disorders. Table 4 presents the proportions of the Australian population in each age and sex category who have anxiety or affective disorders with and without comorbid substance use. The smoking rate within each group is also shown. Among those with comorbid substance use disorders, the smoking rate is consistently high across age and sex group, except for males aged 55 years and over. However, only a minority of those with either anxiety or affective disorders also have comorbid substance use disorders. Although confidence intervals are wide for some groups, smoking rates remain elevated in those without comorbid substance use disorders. Comorbid substance use disorders were most common in younger adults, particularly males.

**Table 3:** Current adult smokers: Proportion with one or more mental disorders in the 12 months prior to the survey, by type of disorder

	US (NCS-R)^a^		Australia (SMHWB)^b^	
	Proportion of current smokers (%)	95% CI	Proportion of current smokers (%)	95% CI

Mental disorders				
Anxiety disorders				
Panic disorder	6.6	5.5 – 7.7	4.6	3.2 – 6.0
Agoraphobia	6.0	4.9 – 7.1	4.7	3.6 – 5.8
Social phobia	10.7	9.1 – 12.3	7.0	5.6 – 8.4
Generalised anxiety disorder	4.9	4.0 – 5.9	5.6	4.0 – 7.2
Obsessive-compulsive disorder	n.a.		3.5	2.2 – 4.8
Post-traumatic stress disorder	7.0	5.6 – 8.3	9.7	7.8 – 11.6
Any anxiety disorder *	22.8	20.2 – 25.4	21.6	18.7 – 24.5
				
Affective disorders				
Depressive episode	5.6	4.5 – 6.7	7.0	5.3 – 8.7
Dysthymia	4.5	3.4 – 5.5	2.2	1.2 – 3.2
Bipolar affective disorder	5.1	4.1 – 6.1	4.6	3.1 – 6.1
Any affective disorder *	12.5	10.9 – 14.1	12.1	9.9 – 14.3
				
Substance use disorders				
Alcohol harmful use	7.2	5.9 – 8.6	5.8	4.2 – 7.4
Alcohol dependence	4.0	2.5 – 5.4	4.0	2.8 – 5.2
Drug use disorder	3.5	2.6 – 4.4	4.7	3.4 – 6.0
Any substance use disorder *	9.6	7.7 – 11.5	12.3	10.2 – 14.4
				
Any mental disorder *	31.7	29.5 – 33.8	32.4	29.5 – 35.3
No mental disorder	68.3	66.2 – 70.5	67.6	64.6 – 70.0
Total smokers	100.0		100.0	

* Note: As a person may have had more than one disorder in the 12 months prior to the survey, the components will not add to the totals shown.

a Source: National Comorbidity Survey – Replication [46].

b Source: 2007 Australian Survey of Mental Health and Wellbeing [43].

**Table 4:** Australian adults 16–85 years: Prevalence of anxiety and affective disorders with and without comorbid substance disorders and smoking rates, by age group and sex

Mental disorder*	Proportion with mental disorder (%)	95% CI	Smoking rate (%)	95% CI
Males aged 18–34 years				
Anxiety with substance use	3.0	1.8 – 4.1	63.4	40.4 – 86.3
Anxiety without substance use	7.5	5.5 – 9.5	33.5	20.6 – 46.5
Affective with substance use	2.2	0.9 – 3.6	72.6	44.5 – 100.0
Affective without substance use	3.5	2.2 – 4.8	46.2	23.9 – 68.5
No 12-month disorder	77.2	73.7 – 80.7	26.5	22.1 – 30.9
Males aged 35–54 years				
Anxiety with substance use	2.7	1.5 – 3.9	79.9	65.7 – 94.1
Anxiety without substance use	11.8	9.0 – 14.5	25.3	15.9 – 34.8
Affective with substance use	1.8	0.8 – 2.8	86.2	72.5 – 100.0
Affective without substance use	5.6	3.8 – 7.5	38.5	22.5 – 54.6
No 12-month disorder	80.2	76.6 – 83.8	24.4	19.8 – 28.9
				
Males aged 55 years and over				
Anxiety with substance use	0.4	0.0 – 0.7	7.8	0.0 – 26.1
Anxiety without substance use	6.3	4.9 – 7.7	22.6	13.9 – 31.2
Affective with substance use	0.1	0.0 – 0.2	95.0	60.0 – 100.0
Affective without substance use	2.0	1.1 – 3.0	25.4	8.0 – 42.9
No 12-month disorder	91.2	89.6 – 92.8	14.7	12.3 – 17.1
				
Females aged 18–34 years				
Anxiety with substance use	2.3	1.4 – 3.2	56.6	39.6 – 73.6
Anxiety without substance use	19.1	17.0 – 21.2	31.4	25.4 – 37.4
Affective with substance use	1.6	0.9 – 2.4	62.4	34.2 – 90.6
Affective without substance use	6.9	5.5 – 8.4	36.4	25.8 – 46.9
No 12-month disorder	71.6	68.9 – 74.3	15.6	13.0 – 18.3
Females aged 35–54 years				
Anxiety with substance use	1.4	0.7 – 2.2	54.0	21.2 – 86.8
Anxiety without substance use	19.8	17.0 – 22.6	36.1	27.0 – 45.3
Affective with substance use	0.6	0.3 – 0.9	75.2	50.9 – 99.6
Affective without substance use	7.5	5.3 – 9.6	45.5	30.9 – 60.1
No 12-month disorder	74.9	71.8 – 78.1	20.1	16.3 – 24.0
				
Females aged 55 years and over				
Anxiety with substance use	0.1	0.0 – 0.2	55.5	20.0 – 85.0
Anxiety without substance use	9.8	8.4 – 11.1	14.8	7.9 – 21.7
Affective with substance use	0.0	0.0 – 0.4	50.0	10.0 – 90.0
Affective without substance use	4.5	3.5 – 5.6	13.3	3.6 – 23.0
No 12-month disorder	87.8	86.3 – 89.3	10.5	8.6 – 12.3

* Note: As a person may have had more than one disorder in the 12 months prior to the survey, the components will not add to the totals shown.

Source: 2007 Australian Survey of Mental Health and Wellbeing [43].

### Use of services

Of the 1 156 600 Australian smokers who had a 12-month mental disorder, as estimated in the SMHWB, 740 000 did not access any health services for their mental health problems in the 12 months prior to the survey (64.0%, 95% CI: 55.4%–72.0%). The smoking rate was not substantively different between those people with 12-month mental disorder who did or did not access services. Of people with 12-month mental disorder who did not use any services, 35.4% were current smokers (95% CI: 31.2%–39.6%), while 37.7% of those with 12-month mental disorder who did use one or more services in the past 12 months were current smokers (95% CI: 32.9%–42.5%). Thus people with 12-month mental disorder who did not have contact with services in the past 12 months represent 20.7% of all current smokers in Australia (95% CI: 18.2%–23.3%).

### Non-specific psychological distress and smoking

**Figure 3:**
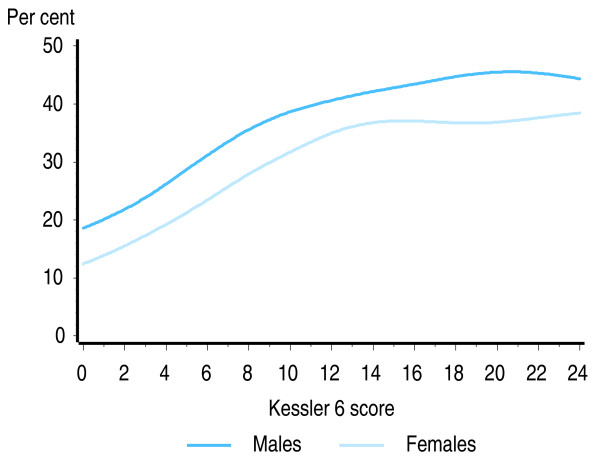
**Proportion of US adult population who smoke, by level of psychological distress and sex**. Source: 2007 United States National Health Interview Survey [49].

Figure 3 shows the relationship between level of psychological distress (measured using the Kessler 6 scale) and smoking rates in the US adult population. Although males have a higher rate of current smoking than females, the shape of the association is the same for both sexes. For both sexes, both the linear and non-linear components of the fitted curves were statistically significant (p < 0.01) indicating both that the increase in smoking rate with increasing level of non-specific psychological distress is significantly greater than zero, and that the deviation of the curve from the linear fit for scores above 13 is statistically significant. On the Kessler 6 scale, scores above 13 are generally considered to represent serious psychological distress likely to require intervention from mental health services.

**Figure 4:**
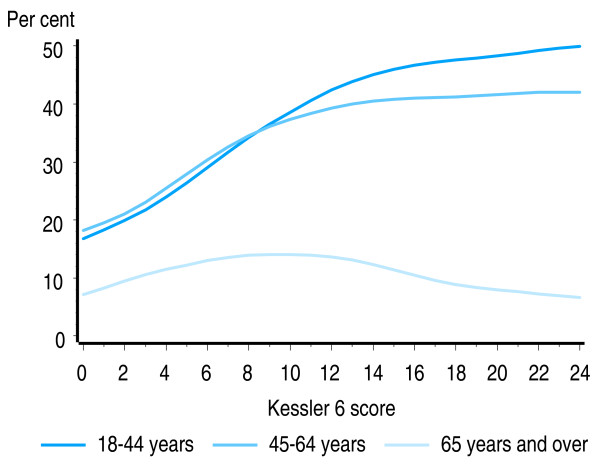
**Proportion of US adult population who smoke, by level of psychological distress and age group**. Source: 2007 United States National Health Interview Survey [49].

By age group, large increases in smoking rates with increasing level of non-specific psychological distress are seen for younger people, with the proportion of smokers increasing from 18% to 50% among people aged 18–44 years, and increasing from 17% to 42% for adults aged 45–64 years. Smaller changes were observed in adults aged 65 and over with the smoking rate peaking at 14% (Figure 4). For all three age groups both the linear and non-linear components of the regression curves were statistically significant (p < 0.01).

**Figure 5:**
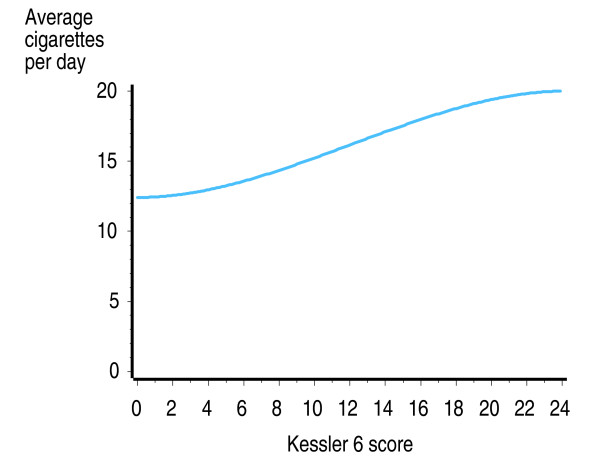
**Current US adult smokers: Average number of cigarettes smoked per day, by level of psychological distress**. Source: 2007 United States National Health Interview Survey [49].

The average number of cigarettes smoked per day by current smokers increased with level of psychological distress, from 12 per day among smokers with no psychological distress, to 19 per day among smokers with serious psychological distress (Figure 5). Both the linear and non-linear components of the regression curve were statistically significant (p < 0.01) indicating that the increase in number of cigarettes smoked by level of psychological distress is unlikely to be attributable to chance alone.

## Discussion

The figures shown here from the latest population surveys in the United States and Australia are consistent with previous reports showing high rates of smoking among adults with mental illness. In the 1997 Australian Survey of Mental Health and Wellbeing, 41% of adults with a 12-month mental disorder were current smokers compared with 21% of adults without a mental disorder, and adults with a 12 month disorder represented 30% of all smokers [57,58]. Changes in the design of the CIDI between 1997 and 2007 mean these figures are not directly comparable with those from the 2007 survey, but the similarity in smoking rates is notable. The 1992 US National Comorbidity Survey found that 41% of adults with a mental illness in the past month were current smokers compared with 22.5% of adults with no lifetime mental illness [11]. In New Zealand, the 2003–04 New Zealand Mental Health Survey reported 33% of adults with a mental disorder were smokers compared with 21% of adults with no mental disorder [59]. In the UK, the 1996 National Psychiatric Comorbidity Survey found 47% of adults with psychiatric morbidity were smokers compared with 29% of those with no psychiatric morbidity [60,61]. Unlike the other surveys, the UK survey used the Clinical Interview Schedule – Revised [62] rather than the CIDI.

Our findings of increasing level of smoking with increasing level of psychological distress are also consistent with previous reports. In the 1992 US National Comorbidity Survey, smokers with a mental illness in the past month smoked a mean 26.2 cigarettes per day compared with 22.6 per day for smokers with no mental illness [11]. In the 2006–07 New Zealand Health Survey, smokers in the top quintile of K10 scores, measuring non-specific psychological distress, smoked 1.21 times as many cigarettes per day as smokers in the lowest quintile [59]. In the 1996 German National Health Interview Survey, smokers with a mental disorder were more likely to meet DSM-IV criteria for nicotine dependence, and more likely to smoke heavily [63].

Although smoking rates have been declining in the US and Australia, our findings demonstrate the continued large proportion of smokers who also suffer from common mental disorders, consistent with our hypothesis. The proportion of people with mental illness who smoke was highest among young adults. As these smokers are likely to have commenced smoking most recently, this suggests the possibility that the proportion of smokers who have a mental illness may rise as these cohorts age.

People with substance use disorders have the highest rates of smoking, and those with anxiety and depressive disorders who have a comorbid substance use disorder also have very high smoking rates. Although this group represents the minority of those with mental illness who smoke, they present particular challenges for tobacco control. Especially among young people, smoking initiation needs to be seen in the context of overall risk-taking behaviour. If a reduction in smoking is offset by an increase in the use of another substance, the benefit of smoking reduction will be reduced.

People with mental illness who are also smokers suffer from the negative health effects of smoking. Mental illness is associated with high rates of morbidity and mortality due to cardiovascular disease, respiratory diseases and cancers [13-17,64,65]. The higher average consumption of cigarettes by smokers with mental illness suggests that mentally ill people who smoke may be even more likely to suffer adverse health consequences than smokers without mental illness. Indeed, the relative success of anti-smoking efforts among the non-mentally ill has been suggested as one reason for the growing disparity in morbidity, mortality and life expectancy outcomes between those with and without mental illness [16,18].

### Implications for tobacco control

The main focus in responding to the high prevalence of mental illness among current smokers has been on interventions aimed at mental health treatment settings, particularly hospital and clinic based settings [66,67]. There is no doubt that the high rate of smoking among patients of mental health services contributes to the excess physical morbidity and mortality consistently observed in these patients, and it is likely that smoking complicates or exacerbates mental illness and its treatment. Efforts to reduce smoking in patients of mental health services are clearly important. However, service-based smoking cessation programmes will be ineffective for the large proportion of smokers with common mental disorders who are not in contact with these services.

Approaches to smoking cessation outside the clinical setting have largely ignored the issue of mental illness. For example, the 2000 US Surgeon General report on reducing tobacco use does not mention this group [68], while the recent Australian National Preventative Health Taskforce report on tobacco control only mentions mental illness outside of mental health services to suggest that people with common mental illnesses are as likely as anyone else to benefit from general population-based smoking cessation strategies [69]. The principal components of population health-based smoking cessation efforts, such as reducing availability, restricting all forms of promotion, increasing price, advertising health consequences and educating young people about them, and restricting use in public places, have generally not been tailored for people with mental illness, and may be less relevant or less effective for people dealing with mental illness. For instance, increases in price may be less successful in motivating quit attempts in people with mental illness. Some of the heaviest smokers have the least ability to pay for their tobacco use [70]. The principle of increasing price to reduce smoking rates is predicated on smokers being rational agents able to maximise utility of available funds. Mental disorders, particularly depression and anxiety disorders can affect people's decision making. It is possible that continuing to increase the price of cigarettes may have a negative effect on mentally ill smokers, penalising both them and their families [37]. Similarly, it has been suggested that bans on smoking in public places may increase the level of social isolation felt by people with mental illness who smoke [38]. Denormalising smoking may be less influential on people who already feel marginalised from mainstream society for other reasons. Without programmes that ensure people with mental illness have as much chance of successfully quitting smoking as anyone else, the success of these types of public policies may further the level of inequality between people with and without mental illness.

Similarly, health promotional messages have concentrated on communicating the longer term negative health consequences of smoking. While this knowledge has prompted some people to quit smoking and prevented others from starting smoking, these messages may have less impact on people suffering mental illness or dealing with stressful and difficult life circumstances. Mental illnesses such as depression or anxiety disorders are associated with increased risk-behaviour, possibly motivated by the lower value placed on a long life by people whose sense of fulfilment in life is compromised [38,71,72]. The ability to focus on longer-term goals and consequences can be affected by the pressures of more immediate and challenging circumstances. There is little evidence in the literature of attempts to develop smoking cessation messages to target issues that are more relevant to people with mental illness.

Population health-based smoking cessation promotional activities have tended to focus on messages for the whole population or segmenting the population by demographic characteristics, such as age and sex. It has been suggested that this focus may reflect the way surveillance data are collected [73], but it is in contrast to the way that cigarettes historically have been marketed in the US and Australia, and the way they continue to be marketed in countries with less restrictions on cigarette marketing and promotion. Analysis of tobacco industry marketing research and market segmentation studies suggests the tobacco industry, in addition to developing products and campaigns targeting youth or women, for example, also targeted market segments defined by psychosocial characteristics, such as personality type. Products and campaigns were developed to address concerns such as reducing anxiety, stress or nervousness, improving mood, increasing social confidence, reducing irritability, or increasing concentration [11,73-76]. Academic studies investigating motivations for taking up and for continuing smoking have also commonly found that psychological factors are important. Coping with stress, and controlling emotions are two factors commonly extracted from these types of studies [77-79]. Smokers are also more likely to deal with problems themselves rather than seek help [77]. As these psychosocial factors cut across demographic groups, demographically targeted smoking cessation campaigns may miss opportunities to develop more effective targeted strategies.

Tobacco control groups have tended to focus on treating smoking as a single issue, arguing that not only are sub-groups such as people with mental illness equally likely to benefit from population-based access control and promotion of long-term health consequences, but that developing targeted campaigns would weaken the effectiveness of the overall campaign [80]. Few would argue with the gains that the universal targeting of smoking cessation has made in the past decades in achieving reductions in the prevalence of smoking. And yet, the evidence here also suggests that substantial inequalities in benefit are accruing in the sub-population of individuals with mental illness and that strategies in addition to universal approaches are needed to address these vulnerable populations.

The universal approach may also miss the context in which young people initiate smoking or partake in some other risk-taking behaviour. A decline in smoking rates would be less meaningful if it is achieved through young people substituting some other harmful substance or behaviour to cope with their difficulties. Perhaps when population health goals are formulated, such as the *Healthy People 2010 *objectives [81], a broader context should be considered, such as reducing smoking related harm without increasing harm through other risk behaviours. As mentally ill smokers may be more susceptible to addictions in general [82], focussing narrowly on the smoking behaviour only without considering other risk behaviours may compromise the overall goal of smoking cessation – improved health outcomes.

### Monitoring smoking in people with mental disorders in routine surveillance systems

The CIDI, which was used for assessing mental health conditions in both the NCS-R and the SMHWB, is time consuming to administer and impractical to incorporate in omnibus surveys of the type that are routinely used for monitoring smoking rates. The K6 measure of non-specific psychological distress has been included in the US National Health Interview Survey since 1997 [52], and has more recently been incorporated into the US Behavioral Risk Factor Surveillance System [83] and the Australian National Health Survey [84]. Although it is far less comprehensive than the CIDI, the strong association between smoking rates and level of psychological distress suggest it could be used to track progress in reducing smoking rates among the mentally ill. As the measure is already incorporated in monitoring surveys, we hope that it can be incorporated into routine surveillance of smoking rates. While some collections have employed a binary categorisation of psychological distress, the significant gradient in smoking rates and numbers of cigarettes consumed across low, mild, moderate, and severe levels of psychological distress suggest at least four or five categories should be employed to adequately monitor this relationship. The K6 scale is most closely correlated with anxiety and affective disorders and less relevant to measuring impulse control or substance use disorders. Its use in routine surveillance should be supplemented by other measures in these areas.

Currently there is no long-term time series on smoking rates in people with and without mental illness. Although the NCS and the SMHWB have both been run twice, the changes in instrumentation and the shift from ICD-9/DSM-III-R to ICD-10/DSM-IV limit the comparability of these figures over time. It is hoped that the inclusion of short mental health measures such as the K6 in routine health surveillance programmes will lead to the accumulation of time series data in this area.

### Study limitations

All three surveys are based on self-report for both mental health status and smoking status. Diagnosis via the CIDI correlates well with diagnosis via structured clinical interview administered by a psychiatrist, but is not the same thing as a validated psychiatric interview. The NCS-R and SMHWB were both voluntary surveys, and response rates may be an issue. It is possible that people with mental illness were under-represented in the surveys due to non-response problems. The surveys targeted the non-institutionalised population, and will thus not include the high rates of mental health problems within institutional settings such as hospitals, hostels, and prisons.

## Conclusion

Data from two major population-based surveys of mental health, in the US and Australia, not only confirm the high rate of smoking among people with mental illness, but also the significant proportion of smokers who have a mental illness. Data from the US NHIS also show that people with higher levels of psychosocial stress on average consume higher numbers of cigarettes.

There may be opportunities to further reduce smoking rates, reduce morbidity and mortality associated with mental illness, and reduce inequality in health outcomes, by specifically targeting some tobacco control efforts at this population of smokers. While mental health services in developed countries have changed considerably in recent years with moves towards more integrated models of care, it seems that providers of some physical health services are more reluctant to see mental illness as an important part of their target group. Groups interested in reducing morbidity and mortality due to heart disease or cancer have traditionally been strong supporters of tobacco control. Despite the disproportionate rate of people with mental illness in morbidity and mortality statistics for these conditions, addressing mental health concerns has not been a high priority for these groups. On the basis of figures reported in major national surveys it would be expected that mental illness would be a key area of concern for groups interested in tobacco control.

It is possible that reducing the incidence of common mental health problems might also reduce the incidence of smoking. Moreover, it is possible that helping people with depression and anxiety problems to find alternative ways of coping, and to understand the consequences of nicotine withdrawal for their symptoms, may be helpful strategies for reducing smoking rates. Teaching young people about mental illness, skills for dealing with emotional difficulties, anxiety and stress, and about the relationship between mental illness, smoking and other risk behaviours in schools and other settings may also help reduce the incidence of smoking. Population-based methods are required as the majority of smokers with mental illness are not in contact with mental health services.

Some people with mental illness may find that quitting smoking may not only improve their physical health, but improve their mental health as well. Market segmentation studies and the development of brands and campaigns for tailored market segments is a common tool of marketing, not just in the tobacco industry. This type of approach may yield gains in the efforts to combat smoking-related harm.

## Competing interests

The authors declare that they have no competing interests.

## Authors' contributions

DL and FM conceived the original idea for the study. All authors contributed to the development of the study methodology. DL acquired and analysed the data, and wrote the first draft of the manuscript. All authors edited the paper. All authors read and approved the final manuscript.

## Pre-publication history

The pre-publication history for this paper can be accessed here:


